# A novel platelet-rich plasma clinically induces reliable, rapid, long-term chronic peripheral neuropathic pain elimination

**DOI:** 10.3389/ebm.2026.10907

**Published:** 2026-03-13

**Authors:** Damien P. Kuffler, Onix Reyes, Ivan J. Sosa, Christian A. Foy

**Affiliations:** 1 Institute of Neurobiology, Medical School, University of Puerto Rico, San Juan, PR, United States; 2 Doctor’s Center Hospital, Manati, PR, United States; 3 Section of Neurosurgery, Medical School, University of Puerto Rico, San Juan, PR, United States; 4 Section of Orthopedic Surgery, Medical Sciences Campus, University of Puerto Rico, San Juan, PR, United States

**Keywords:** autografts, chronic neuropathic pain, nerve regeneration, nerve trauma, PRP

## Abstract

Peripheral nerve trauma results in 50%–84% of patients developing chronic neuropathic pain, which is eliminated when axons reinnervate targets. Autografts reduce pain by promoting target reinnervation. We hypothesized that applying a novel platelet-rich plasma (PRP) formulation to proximal stumps would permanently eliminate the pain. This prospective case series compared analgesia levels after bridging nerve gaps with an autograft (autograft repair) vs. a PRP-filled collagen tube (PRP repair). Autograft repairs were performed on 16 nerves with a 5.75-cm mean gap length, 2.0-year repair delay, 42.3-year age, and 8.6 chronic neuropathic pain. PRP repairs were performed on 10 nerves with a 6.0-cm gap length, 1-year repair delay, 36.7-year age, with 88% having 9.1 chronic neuropathic pain. For autograft repairs, the pain began to decrease when axons reinnervated targets, reaching a mean of 0.3 in 18.2% of patients, and was eliminated in 81.8%. Following PRP repairs, the pain reduction began within 2 weeks and was eliminated by 2 months. Thus, autografts contribute to pain reduction/elimination by promoting target reinnervation. However, PRP directly and rapidly induced long-term pain elimination in all patients, while axons were regenerating, and without target reinnervation. These results prove that platelet-released factors reliably and rapidly eliminate chronic neuropathic pain.

## Impact statement

Peripheral nerve trauma results in up to 85% of patients suffering chronic neuropathic pain, which can have a profound negative impact on patients’ lives because no technique induces reliable or rapid long-term pain elimination. This study shows that applying PRP of a novel composition to nerves evoking chronic pain reliably and rapidly eliminated the pain in each patient, regardless of age, repair delay, and lack of target reinnervation. The acceptance and broad application of this novel PRP to nerves evoking chronic neuropathic pain may eliminate chronic neuropathic pain in all patients.

## Introduction

Peripheral nerve trauma not only eliminates sensory and motor function but also results in 50%–84% of individuals developing chronic neuropathic pain [1]. The first line of treatment for chronic neuropathic pain is pharmacological agents. While they may reduce/eliminate pain, their analgesic effects are limited, short-lived, and may cause severe side effects that preclude their use.

A secondary approach for restoring function and reducing chronic neuropathic pain is to use sensory nerve autografts, the clinical “gold standard” surgical technique, to bridge nerve gaps to restore function. Although autografts do not directly reduce or eliminate pain, they contribute to pain reduction/elimination by promoting axonal regeneration across the graft and into the distal part of the nerve, leading to target reinnervation, which, in turn, reduces/eliminates the pain [2]. The present case series was designed to determine whether, compared to autografts, bridging nerve gaps with a PRP-filled collagen tube results in greater long-term reduction or elimination of chronic neuropathic pain, while also restoring meaningful sensory and motor recovery. PRP has been shown to enhance axon regeneration and reduce pain [3]. The efficacy of PRP promoting recovery is discussed in a separate paper, while this paper focuses on PRP and its ability to eliminate chronic neuropathic pain.

## Materials and methods

### Research objectives

Determine the relative efficacy of bridging peripheral nerve gaps with an autograft vs. a PRP-filled collagen tube in reducing/eliminating chronic neuropathic pain and the duration of the pain suppression.

### Hypothesis

Platelet-released factors induce long-term chronic neuropathic pain reduction/elimination.

### Research subjects

Patients presenting to the Department of Orthopedic Surgery service who required a peripheral nerve gap repair.

### Inclusion criteria

Subjects aged 18–75 years old with a nerve gap that required repair. Each exhibited one or more of the following three conditions that are known to reduce or prevent recovery: A gap larger than 5 cm, nerve repair delay more than 5 months post-injury, and age more than 25 years.

### Experimental design

This formal prospective consecutive study treated the PRP repair individuals as experimental subjects, followed by the retrospective analysis of control autograft repair patients. The study compared the level of chronic neuropathic pain pre- and post-surgery following upper extremity nerve gap repairs using an autograft vs. a PRP-filled collagen tube.

### Randomization

No.

### Blinding

No blinding.

### Sample size

Power analysis indicated that the study required a sample size of 8 patients to achieve a significance level of p < 5%.

## Surgery

Under full anesthesia, nerve injury sites were exposed, the damaged nerve tissue cut away with a scalpel, and the nerve stumps refreshed under a microscope, where clear nerve fascicles were seen in the proximal nerve stump, and no scarring was seen in the distal stump/s.

The resulting nerve gap/s were measured.

### Collagen tubes

Before the availability of FDA-approved collagen tubes, collagen tubes were made using FDA-approved 2 × 8 cm collagen sheets (Veritas, Synovis Life Technologies, St. Paul, MN). The tubes were created by sewing a sheet around the handle of a surgical tool as a template. Tubes from 8 cm sheets can be used to repair gaps ≤7.6 cm in length, allowing the autograft and nerve stump coaptation sites to be 1–2 mm inside the PRP-filled collagen tube. To bridge longer gaps, two collagen sheets were sewn together end-to-end and into a tube. Subsequently, commercially available NeuroMend Collagen tubes (Regenity Biosciences, Oakland, NY) were used.

### Securing the nerve stumps within the collagen tube

Autografts were loosely secured with a single suture to the proximal and distal nerve stumps.

### Preparation of platelet-rich fibrin

Under general anesthesia, but before any surgical intervention, 55 cc of whole blood were drawn from a peripheral vein into a 60-cc syringe containing 5 cc of citrate-based anticoagulant. The PRP was separated using Gravitation Platelet Separation III (GPS III) centrifuge tubes (Zimmer Biomet Corp., Warsaw, IN), yielding ca. 6 cc of PRP.

### Filling the collagen tube with PRP

**FIGURE 1:**
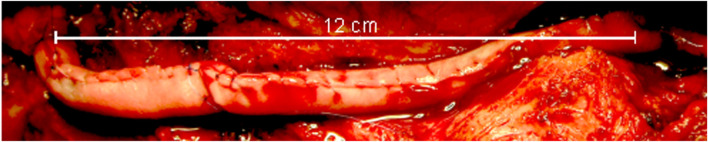
Repaired and PRP-filled 12-cm nerve gap. Two collagen sheets were sewn end-to-end, then into a tube, before being injected with PRP.

The PRP was drawn from the GPS centrifuge tube into a 10 mL syringe, while 0.6 cc of thrombin was drawn into a 1 cc syringe. The syringes were attached to a FibriJet Ratio Applicator Assembly SA-1001 with an attached flexible blending connector SA-3673 (Nordson Medical, Westlake, OH). The catheter was inserted into the collagen tube, and both syringe plungers were pressed simultaneously, mixing and injecting the syringe contents into and filling the collagen tube. The fibrin polymerized within 20 s, and the patient closed. Figure 1 shows the completed repair of a 12-cm-long nerve gap in a 58-year-old patient, which was repaired 3.25 years after nerve trauma.

### Rules for stopping data collection

The original data collection was intended to stop when the last enrolled patient was 1.5 years post-surgery. However, the data of one patient, collected 10 months post-surgery, are included because he disappeared shortly after the electrophysiological and physical tests were performed.

### Patient follow-up

To ensure consistency in the electrophysiological and physical studies and analyses, they were performed on all patients by the same clinicians within a few-month period.

### Pain evaluation

Each patient self-evaluated their neuropathic pain level before and after surgery using the validated 11-point qualitative pain assessment linear pain scale, ranging from 0–10, where 0 = no pain and 10 = worst pain possible.

### Statistical analysis

Statistical analysis involved using Excel to calculate means and standard deviations, and regression analysis was used to examine relationships among the variables.

### Adverse events

No patient experienced any adverse events, such as infections or worsening or new neuropathic pain.

### Ethical practice

This study was performed under a local IRB-approved protocol and in accordance with the World Medical Association Declaration of Helsinki (JBJS 79-A:1089-98,1997).

### Informed consent

Each recruited patient signed an IRB-approved Written Consent Form.

### Patient gender

Patients were enrolled as they presented, without consideration of gender.

## Results

### Demographics

The autograft repairs involved 16 upper extremity nerves in 11 patients, and the PRP repairs involved 10 upper extremity nerves in 8 patients. For the autograft and PRP repairs, the mean gap lengths were 5.75 and 6.0 cm, mean repair delays 2.0 and 1.0 years, respectively, and mean patient ages 42.3 and 36.7 years, respectively**.** Table 1 all the nerves were mixed sensory and motor, with 37.5% median and 56.3% ulnar. The gap locations were 12.5% elbow, 50% forearm, and 37.5% wrist.

**TABLE 1:** Demographics and pain outcomes.

Descriptor	Autograft repairs	PRP repairs	% Difference
# Patients	11	8	​
# Nerves	16	10	​
Female/male	1/10	0/8	​
Gap length	2–8 cm (mean 5.75 ± 2.05 SD)	2–12 cm (mean 6 ± 2.6 SD)	4.3% longer
Repair delay	0.25–6.3 years (mean 2.01 ± 0.59 SD)	2 weeks - 3.25 years (mean 1 ± 1.1 SD)	2-fold longer
Age	19–71 years (mean 42.3 ± 16.3SD	18–58 years (mean 36.7 ± 13.7 SD)	13.3% older
Patients with pre-surgery pain	100%	88%	12% fewer
Pre-surgery pain range	4–10	7–10	​
% Patients with pain of 8–10	81.8%	86%	​
Mean pre-surgery pain	8.6 ± 4.2 SD	9.1 ± 1.2 SD	​
Post-surgery follow-up time range	1.1–3 years (mean 1 ± 0.7 SD)	10 months - 17.8 years (mean 13.3 ± 0.4 SD)	​
Post-surgery pain range	0–2	0	​
Mean post-surgery pain	0.3 ± 0.3 SD	0	​
Patients with pain reduced	18.2%	--	​
Patients with pain eliminated	81.8%	100%	​

### Pain reduction/elimination

Before surgery, 100% of the autograft repair patients and 88% of the PRP repair patients suffered chronic neuropathic pain, respectively, with 81.8%and 86% having a mean pain of 8.6 vs. 9.1, respectively. Table 1 For autograft repairs, pain began to decrease around the time axons started to reinnervate targets, reaching a mean of 0.3 in 18.2% of patients, and was eliminated in 81.8% of patients. For the PRP repairs, each patient’s pain began to decrease within 2 weeks of surgery and was eliminated within 2 months.

## Discussion

### Reducing chronic neuropathic pain: pharmacological agents and surgery

Peripheral nerve trauma leads to 50%–85% of patients suffering chronic neuropathic pain [1]. The level of pre-operative neuropathic pain is a good predictor of neuropathic pain persisting after the nerve repair [4]. Furthermore, increasing repair delays are associated with smaller reductions in post-repair chronic neuropathic pain [5].

Pharmaceutical agents, such as gabapentin, provide adequate pain control to only 30%–40% of patients, but they do not eliminate the pain. Further, they often induce debilitating and intolerable side effects that preclude their use.

More effective than pharmaceuticals in reducing neuropathic pain are autografts and allografts, the clinical “gold standard” techniques for repairing nerve gaps. They induce similar amounts of target reinnervation and meaningful recovery, which, in turn, leads to reliable chronic neuropathic pain reduction/elimination in <50% of patients [6].

It is hypothesized that neuropathic pain remains chronic as long as axons are regenerating and is only reduced/eliminated when axons reinnervate targets, stop regenerating [2], and pick up target-derived factor/s that stop the nociceptive axon hyperactivity that underlies the pain [7]. This hypothesis led to the development of targeted muscle reinnervation (TMR) and regenerative peripheral nerve interface (RPNI) techniques that reduce or eliminate chronic neuropathic pain.

In animal models [8] and clinically [9], TMR and RPNI reduce the development of pain-inducing neuromas, thereby reducing neuroma pain in 75%–100% of patients, and reducing both post-amputation and phantom limb pain (PLP) in 45%–80% [9]. However, TMR and RPNI have the limitations that their analgesic efficacy decreases when applied >3 months post-trauma [10]. Further, they cannot be used if one’s goals are to reduce pain and restore meaningful sensory and motor function.

### PRP efficacy in reducing pain

Animal model studies show that PRP reduces chronic pain behavior associated with *Mycobacterium leprae* (leprosy bacteria) induced lesions [11], and when applied to sites of skin burn-induced neuropathic pain [12], and rat spinal cord injury sites [13]. Clinically, chronic neuropathic pain is reduced by applying PRP to sciatic [14] and digital nerve crush sites [15], neurolysis surgery sites [16], when applied to nerves during carpal tunnel surgery [17], following PRP intraneural injection, when injected into the intervertebral disc epidural space [18], and into the perineurium of patients suffering from diabetic neuropathic pain [19]. A single PRP injection provides analgesia against lower back pain lasting up to 2 years [20]. However, despite multiple PRP injections, the analgesia was consistently incomplete and temporary, and about one-third of patients got no pain relief [20]. However, a recent case series showed that bridging nerve gaps with an autograft within a PRP-filled collagen tube resulted in reliable and rapid long-term reduction of chronic neuropathic pain in 11% of patients and complete pain elimination in 89% of the patients [3].

The present study compared the effect of bridging nerve gaps with an autograft vs. a PRP-filled collagen tube on chronic neuropathic pain levels. Before surgery, all the autograft repair patients suffered chronic neuropathic pain of 8.6, with 81.8% of 8–10. Each patient’s pain began to decrease when axons started to reinnervate their targets, and was reduced to a mean of 0.3. Before the PRP repairs, 88% of the patients suffered a mean neuropathic pain of 9.1, with 86% of 8–10. Following surgery, each patient’s pain began to decrease within 2 weeks, and was eliminated in all the patients within 2 months. Thus, the pain was eliminated while the axons were regenerating and before they had innervated any targets, which demonstrates that platelet-released factors alone can induce long-term chronic neuropathic pain reduction/elimination.

The greatly varied analgesic efficacy of PRP in different studies raises the question of why the PRP used in the present study was far more effective in inducing long-term pain elimination than other PRP. We propose that this was due to the manner in which the PRP was prepared and applied. First, commercial and non-commercial preparation systems yield PRP that varies significantly. These differences include major variations in blood components, glucose levels, pH, platelet concentration, percentage of activated vs. unactivated platelets, bioactive vs. non-bioactive factors, and the presence of other cell types, such as leukocytes and red blood cells. The PRP platelet concentration in this study was increased 9.3-fold, approximately 2-3-fold over PRP from other sources, such as following single-spin separation, which yields a 2.7-fold increase in platelet concentration, or double-spin, which yields PRP with a 2.5 - 5.7-fold increased platelet concentration, with PRP efficacy increasing with increasing platelet concentration [21].

Second, the PRP leukocyte concentration was increased 5-fold (Data on file at Biomet Biologics, LLC) compared to other PRP. While leukocytes can induce inflammation, they also exert anti-inflammatory and analgesic effects [22]. Third, most studies use a small PRP volume (<1 cc), whereas the present study used 4–6 cc. Fourth, while most studies merely apply PRP to the surface of nerves, the present study surrounded the PRP with a collagen tube, which, in rats, increases PRP efficacy [14].

In the present study, PRP eliminated the pain in each patient. In contrast, a larger study might find variability in the extent of pain reduction/elimination among the patients. These differences are best explained by variations in the patient’s whole blood platelet concentration, which can vary up to 2-fold 23, and by physiological factors, such as diet, medications, and health issues [23].

### Potential mechanisms of PRP action

Nerve injury induces nociceptive neurons to upregulate Nav1.8 sodium channel expression, leading to axon hyperexcitability, neuropathic pain, painful neuropathies, and inflammatory pain [24]. This makes the Nav1.8 channel a good target for reducing/eliminating chronic neuropathic pain. PRP may also reduce/eliminate pain by platelet-released transcription factor 4 (TCF4), down-regulating Nav1.8 channels [25], thus silencing chronically active nociceptive axons.

Among alternative mechanisms by which platelets may reduce pain is by their induction of monocytes to release the anti-inflammatory cytokine IL-10, which can reduce pain in several ways. First, by suppressing the production of pro-inflammatory cytokines and increasing the expression of anti-inflammatory cytokines [26]. It can also act directly on neurons [27] by down-regulating Nav1.6 and Nav1.8 [28], which contribute to chronic neuropathic pain. Second, platelets can reduce neuropathic pain by releasing TNF-α [29].

## Conclusion

Before surgery, all autograft repair patients suffered a mean chronic neuropathic pain of 8.6. Which was reduced to a mean of 0.3, with 18.2% experiencing long-term pain reduction, and 81.2% long-term pain elimination. For the PRP repairs, before surgery, before surgery, 88% of the patients suffered chronic neuropathic pain of 9.1, which was eliminated in all the patients. For the autograft repairs, the pain began to decrease about the time the regenerating axons started reinnervating their targets. For the PRP repairs, their pain began to decrease within 2 weeks and was eliminated within 2 months. This elimination occurred while the axons were regenerating and long before any regenerating axons innervated targets. The extent of pain reduction/elimination was not influenced by higher pre-surgical pain levels or the increasing values of the independent variables of gap length, repair delay, and patient age. In conclusion, this study proves that platelet-released factors reliably induce long-term chronic neuropathic pain elimination within 2 months, while axons are regenerating, and without requiring target innervation.

### Study limitations

The primary limitation of this study is its small sample size, although it was adequate for statistical analysis.

## Data Availability

The raw data supporting the conclusions of this article will be made available by the authors, without undue reservation.
